# Virus Adaptation and Selection Following Challenge of Animals Vaccinated against Classical Swine Fever Virus

**DOI:** 10.3390/v11100932

**Published:** 2019-10-10

**Authors:** Ulrik Fahnøe, Anders Gorm Pedersen, Camille Melissa Johnston, Richard J. Orton, Dirk Höper, Martin Beer, Jens Bukh, Graham J. Belsham, Thomas Bruun Rasmussen

**Affiliations:** 1 National Veterinary Institute, Technical University of Denmark, Lindholm, DK-4771 Kalvehave, Denmark; ulrik@sund.ku.dk (U.F.); camille.johnston89@gmail.com (C.M.J.); grbe@sund.ku.dk (G.J.B.); 2 Department of Health Technology, Section for Bioinformatics, Technical University of Denmark, DK-2800 Kgs. Lyngby, Denmark; agpe@dtu.dk; 3 Copenhagen Hepatitis C Program (CO-HEP), Department of Infectious Diseases, Hvidovre Hospital and Department of Immunology and Microbiology, Faculty of Health and Medical Sciences, University of Copenhagen, DK-2200 Copenhagen N, Denmark; jbukh@sund.ku.dk; 4 Institute of Biodiversity, Animal Health, and Comparative Medicine, College of Medical, Veterinary and Life Sciences, University of Glasgow, Glasgow G12 8QQ, UK; richard.orton@glasgow.ac.uk; 5 Medical Research Council-University of Glasgow Centre for Virus Research, Institute of Infection, Inflammation and Immunity, College of Medical, Veterinary and Life Sciences, University of Glasgow, Glasgow G61 1QH, UK; 6 Institute of Diagnostic Virology, Friedrich-Loeffler-Institut, 17493 Greifswald-Insel Riems, Germany; dirk.hoeper@fli.de (D.H.); martin.beer@fli.de (M.B.)

**Keywords:** vaccination, virus evolution, classical swine fever virus, CSFV, virulence, deep sequencing, viral populations, haplotype selection

## Abstract

Vaccines against classical swine fever have proven very effective in protecting pigs from this deadly disease. However, little is known about how vaccination impacts the selective pressures acting on the classical swine fever virus (CSFV). Here we use high-throughput sequencing of viral genomes to investigate evolutionary changes in virus populations following the challenge of naïve and vaccinated pigs with the highly virulent CSFV strain “Koslov”. The challenge inoculum contained an ensemble of closely related viral sequences, with three major haplotypes being present, termed A, B, and C. After the challenge, the viral haplotype A was preferentially located within the tonsils of naïve animals but was highly prevalent in the sera of all vaccinated animals. We find that the viral population structure in naïve pigs after infection is very similar to that in the original inoculum. In contrast, the viral population in vaccinated pigs, which only underwent transient low-level viremia, displayed several distinct changes including the emergence of 16 unique non-synonymous single nucleotide polymorphisms (SNPs) that were not detectable in the challenge inoculum. Further analysis showed a significant loss of heterogeneity and an increasing positive selection acting on the virus populations in the vaccinated pigs. We conclude that vaccination imposes a strong selective pressure on viruses that subsequently replicate within the vaccinated animal.

## 1. Introduction

RNA viruses have some of the highest mutation rates known in nature [1]. This allows them to adapt quickly to selective pressures such as host immune responses. Classical swine fever virus (CSFV), an RNA virus belonging to the genus *Pestivirus* within the Flaviviridae family [2], also displays this characteristic. CSFV is the causative agent of classical swine fever (CSF) and exists as multiple genotypes with varying phenotypes ranging from high to low virulence [3,4]. Studies on CSFV have revealed that highly virulent viruses have higher sequence diversity compared to viruses of lower virulence [5]. Whether this high diversity is necessary for high virulence is not fully understood [6,7]. However, high diversity and quasispecies development have been linked to virulence and tissue tropism in picornaviruses [8,9]. The ability of CSFV to adapt quickly during virus replication has been observed in modified, live, attenuated vaccine-viruses in which key changes revert to their parental state after a few passages in cell culture [10]. A study of CSFV adaptation in vivo of another live, attenuated vaccine strain (GPE-) also found the reversion of key motifs after extensive passaging in pigs resulting in a more virulent form [11]. Furthermore, evolution to higher virulence occurred within animals infected with a mutant (and less virulent) form of the usually highly virulent CSFV strain “Koslov” [7]. Adaptation under high selective pressure (such as during antiviral treatment, in the presence of neutralizing antibodies or following vaccination) has the potential to lead to the selection of adaptive escape variants. Examples of this process have been described in vivo and in vitro with the hepatitis C virus (HCV) [12,13,14,15] and in vitro for CSFV [16]. 

Vaccination studies typically focus on the efficacy and safety of the CSF vaccine candidates [17]. However, vaccinated animals often show low-level and transient viral RNA loads after a subsequent virus challenge [18,19,20,21,22]. This indicates that some replication of the challenge virus occurs under the strong selective pressure imposed by the immune system. We have here undertaken a detailed analysis of the virus subpopulations present during this transient period of viremia, in order to analyse the evolutionary processes taking place. Further exploration of evolutionary events in vaccinated animals should facilitate a better understanding of the adaptive potential of the challenge virus and thereby the protective capabilities of vaccine candidates. Next-generation sequencing (NGS) technologies have made it possible to study the evolution of virus populations in great detail. In particular, the use of deep sequencing allows for the identification of low-frequency single nucleotide polymorphisms (SNPs) in virus populations, something that has previously been possible only by end-point limiting dilution or extensive cDNA cloning.

In this study, full-genome sequencing of the challenge virus was performed on samples obtained from pigs that were first inoculated with one of two different live attenuated CSF vaccine candidates and subsequently challenged with the highly virulent CSFV strain “Koslov”. Deep sequencing allowed detailed analyses of the virus populations present within the challenge inoculum and within both naïve and vaccinated animals post-challenge.

## 2. Materials and Methods

### 2.1. Vaccine and Challenge Virus

CSFV C-strain vaccine vR26 and the chimeric derivative vR26_E2gif [10], with vR26_E2gif having the complete E2 sequence from border disease virus (BDV) strain “Gifhorn” [23], were used for the vaccination of pigs. Blood from a pig infected with the highly virulent CSFV strain “Koslov” (CSFV/1.1/dp/CSF0382/XXXX/Koslov) was used as the challenge inoculum [20].

### 2.2. Vaccination and Challenge Infection of Animals

A vaccination/challenge experiment including 21 pigs was performed to assess the vaccine properties of vR26 and vR26_E2gif [10]. In brief, 2 groups of 6 pigs (p1-p6 and p10-p15; 6 weeks old), in separate pens, were vaccinated intramuscularly with either vR26_E2gif (2 × 10^7.7^ TCID_50_) or vR26 (2 × 10^6.9^ TCID_50_). Each vaccine batch was prepared from the 12th passage in SFT-R cells [10]. Further, 3 additional pigs in each group (p7–p9 and p16–p18) served as sentinel animals. A further 3 animals (p19–p21), housed in a separate pen, were mock-vaccinated with cell culture medium and served as the challenge control group (termed naïve). Four weeks later (post-infection day (PID) 0), all pigs, except for the sentinels, were challenged, by the oronasal route, with the CSFV strain “Koslov” (2 × 10^6^ TCID_50_). Clinical signs and rectal body temperature were monitored on a daily basis and scored as previously described [24]. Blood samples and nasal swabs were examined for viral RNA load using quantitative RT-PCR (RT-qPCR) assays and serum samples were tested for anti-CSFV E2 antibodies by ELISA [7,18]. All experimental procedures, including euthanasia, animal care, and maintenance were conducted in accordance with Danish and EU legislation (Consolidation Act 474 15/05/2014 and EU Directive 2010/63/EU) and with the approval from the Danish Animal Experimentation Inspectorate (license number 2008/561-1541).

### 2.3. RNA Extraction and NGS

RNA was extracted from selected samples using a modified Trizol/RNeasy protocol as previously described [25]. Briefly, RNA was extracted from the challenge inoculum, blood, and serum samples using Trizol LS (Invitrogen, Carlsbad, USA), the aqueous phase containing the RNA was applied to an RNeasy spin column (Qiagen, Hilden, Germany) and RNA was eluted in nuclease-free H_2_O. For RNA extraction from tonsils, the tissue was cut into small pieces with sterile scissors. A 100 mg of tissue was added to Trizol LS (750 μL) and H_2_O (250 μL) in a 1.5 mL tube. One steel ball was placed in each tube and processed in a Tissuelyser (Qiagen) at 25 Hz for 1 min. RNA from the resultant homogenate was then extracted as above. The CSFV RNAs from the virus inoculum, blood, and tonsils, with similarly high CSFV RNA levels, were amplified as cDNA by full-length RT-PCR as described [7], whereas the RNA from pigs with low viral loads, where full-length amplification failed, was amplified as two overlapping cDNA fragments (each approximately 6 kbp) using a modified protocol. This modified protocol used an RT step primed by two specific primers (CSF-Kos-6240-RT 5′ TCT ATA GGG TGT TTC TGC CC 3′ and 3′CSF-kos_rev-RT 5′ GGG CCG TTA GGA AAT TAC CTT AGT 3′ [26]. Subsequent amplification of the cDNA was achieved within two separate PCRs to cover the entire genome (the primers used were: 5′ end: CSF-Kos_Not1-T7-1-59 5′TCT ATA TGC GGC CGC TAA TAC GAC TCA CTA TAG TAT ACG AGG TTA GTT CAT TCT CGT ATG CAT GAT TGG ACA AAT CAA AAT TTC AAT TTG G 3′ [7] with CSF-Kos-6176-R 5′ CTG GTG TTG CGG TCA TGG CTA CTA C 3′) and (3′ end CSF-Kos-5981-F 5′GGG GAG ATG AAA GAA GGG GAC ATG 3′ with CSF-kos_12313aR 5′ GGG CCG TTA GGA AAT TAC CTT AGT CCA ACT GT 3′ [26]). The two fragments were pooled in equal amounts (2 × 250 ng) and sequenced (see below). RNA extracted from blood by MagnaPure extraction (Roche, Basel, Switzerland), as used for the RT-qPCR, was also amplified with this modified protocol. In order to get adequate amounts of these RT-PCR products from tissues (in this case tonsils) that contain high levels of host RNA, a modified RNA extraction was applied. The aqueous phase from the Trizol extraction containing the RNA was fractionated on an RNeasy column, five separate eluates were analysed using full-length RT-PCRs. The strongest and clearest products corresponding to the full-length CSFV cDNA were generated from fractions 3 to 5 (Table S1).

NGS was performed on both the RT-PCR products and on RNA using the Ion Plus fragment library kit (Life Technologies, Carlsbad, USA) and sequenced by the Ion PGM platform (Life Technologies) using the SPRIworks—Fragment Library System II (Beckman Coulter, Krefeld, Germany) for the FLX platforms (Table S2).

### 2.4. Analysis of Sequence Data

FastQC [27] was applied for pre-trimming evaluation of the Fastq files containing the raw sequence reads. Trimming and primer removal were performed using cut-adapt and prinseq-lite [28]. Fastq files were error-corrected using RC454 [29] with the CSFV strain “Koslov” nucleotide sequence (GenBank HM237795; [26]) as reference. The de novo assembly was performed using Newbler 2.6 (Roche software, Basel, Switzerland). Each error-corrected Fastq file was aligned using the BWA-MEM [30] algorithm or Mosaik [31]. Subsequently, the libraries were post-processed with Samtools [32] and SNPs were called using lofreq [33]. Downstream SNP effect analysis was performed with snpEff [34] with no fraction cut-off set. SNP plots were made in R. dN/dS and πN/πS analyses were performed by taking the filtered SNP calls and running them through SNPGenie [35] with a SNP cut-off set at 1%. Statistical analysis was performed with Graphpad Prism using the T-test with Holm–Sidak correction for multiple comparisons when appropriate. 

## 3. Results

### 3.1. Vaccinated Pigs Are Protected against Challenge with CSFV but Display Transient, Low Levels of Viral RNA in Blood

**Figure 1:**
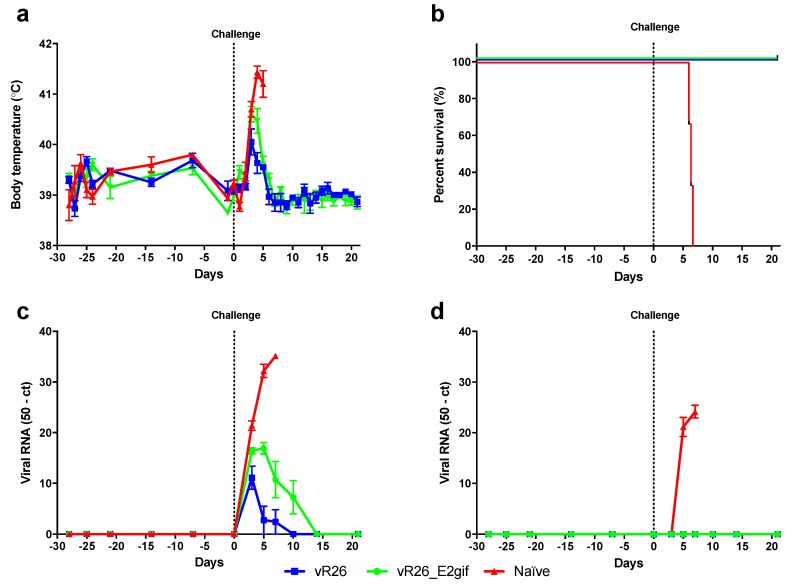
Naïve pigs (*n* = 3) or pigs vaccinated with vR26 (*n* = 6), or vR26_E2gif (*n* = 6) were challenged with the highly virulent classical swine fever virus (CSFV) strain “Koslov”. The graphs show the (mean ± SEM values where appropriate) for: (**a**) body temperature, (**b**) pig survival (%), (**c**) viral RNA in blood, measured by RT-qPCR, shown as 50-ct, and (**d**) viral RNA in nasal swabs measured by RT-qPCR.

All pigs in the naïve (unvaccinated) group, that were challenged with the highly virulent CSFV strain “Koslov”, developed a high fever shortly after challenge (Figure 1a) and were euthanized due to severe clinical signs of CSF within 6 days (Figure 1b). High viral loads were observed in blood and nasal swabs (Figure 1c,d). Pigs previously vaccinated with the CSFV C-strain vR26 and the chimeric vR26_E2gif [10] all survived the viral inoculation that lead to 100% mortality in the naïve pigs (Figure 1b). Transient fever was observed in the vaccinated animals after the challenge, with the highest temperatures seen in the vR26_E2gif vaccinated pigs (Figure 1a). Low levels of viral RNA were observed, between PID3 and PID10, in blood samples from the vR26_E2gif vaccinated pigs, whereas vR26 vaccinated pigs showed lower viral RNA levels between PID3 and PID7 post-challenge (Figure 1c). None of the vaccinated pigs had detectable levels of viral RNA in the nasal swab samples collected post-challenge (Figure 1d). 

The vaccine virus was not detectable in blood at any time before challenge (Figure 1c,d). Furthermore, all pigs vaccinated with vR26 seroconverted against the CSFV E2 within 21 days of the vaccination, whereas pigs vaccinated with the chimeric vR26_E2gif (that expresses the BDV E2 protein) only seroconverted against CSFV E2 after the Koslov challenge (Figure S1). Transmission of the CSFV vaccine or challenge virus to sentinel pigs was not observed; they all remained negative by RT-qPCR throughout the experiment and did not seroconvert.

### 3.2. Deep Sequencing of Virus in the Challenge Inoculum Shows a Heterogeneous Population with a Consensus Sequence Identical to the Koslov Reference

**Figure 2:**
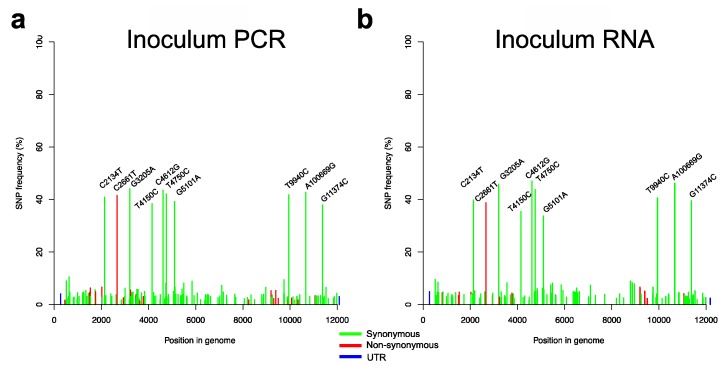
Single nucleotide polymorphism (SNP) frequency plots of the challenge virus in the inoculum. SNPs with a frequency above 20% are indicated for each sample type as follows: (**a**) Virus inoculum (determined following RT-PCR), and (**b**) virus inoculum (determined directly from the RNA). The red, green and blue colours of the bars represent non-synonymous, synonymous and untranslated region (UTR) mutations, respectively.

The virus inoculum used for the challenge infection was deep-sequenced from full-length RT-PCR amplicons and directly from the RNA (Figure 2a,b). The sequencing of the amplicons showed the consensus to be 100% identical to the reference sequence for the CSFV strain “Koslov” (Genbank HM237795). However, variant calling showed that the underlying viral population is in fact quite heterogeneous (Figure 2a) with 10 SNPs having frequencies of about 40%. One of these is a non-synonymous change in the E2 protein, C2661T corresponding to the amino acid change S763L (residue 74 in the E2 protein) that is situated on the surface of domain 1 in the antigenic region B/C, whereas the other nine SNPs are all synonymous (C2134T, G3205A, T4150C, C4612G, T4750C, G5101A, T9940C, A10669G, and G11374C). All other SNPs detected (149 in total) had frequencies between 1% and 10% with the majority corresponding to synonymous mutations. Direct sequencing of viral RNA from the inoculum confirmed these results by having a similar SNP profile (Figure 2b).

### 3.3. Analysis of CSFV Sequences in Naïve and Vaccinated Animals Following Challenge with the Koslov Strain

**Figure 3:**
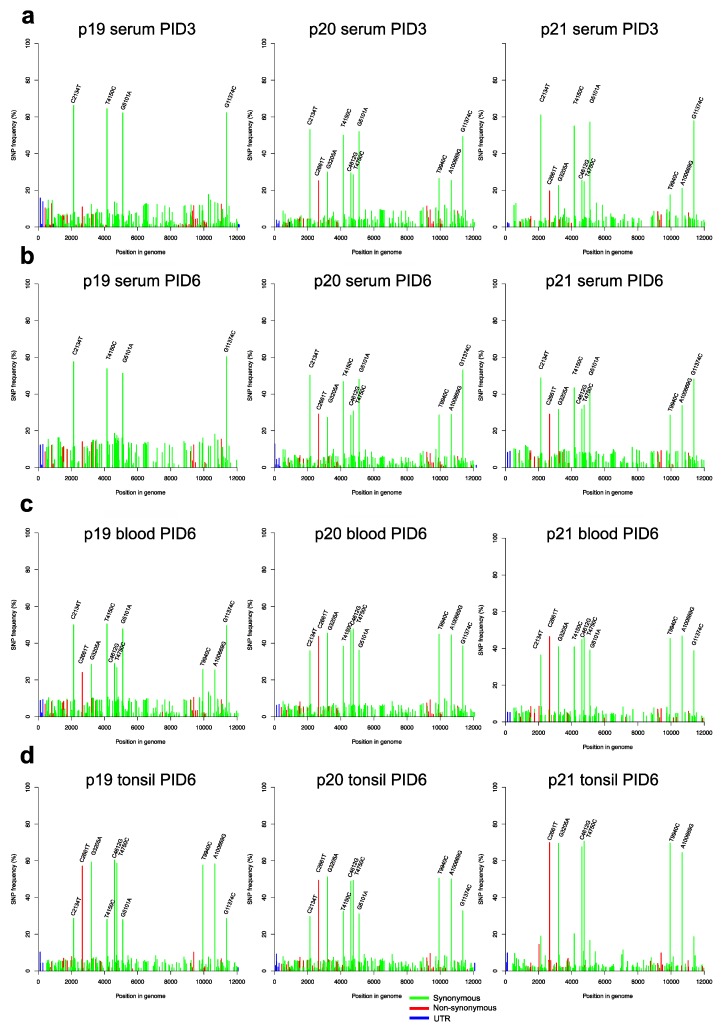
SNP frequency plots of the virus in the naïve pigs (p19, p20, and p21 respectively) following virus challenge. SNPs present at a frequency above 20% are indicated on each plot in the following samples: (**a**) serum (PID3), (**b**) serum (PID6), (**c**) blood (PID6), and (**d**) tonsils (PID6). The SNP frequency plots for serum (PID6) were determined directly from RNA, whereas the others were determined following RT-PCR. The red, green, and blue colours of the bars represent non-synonymous, synonymous, and untranslated region (UTR) mutations, respectively.

**Figure 4:**
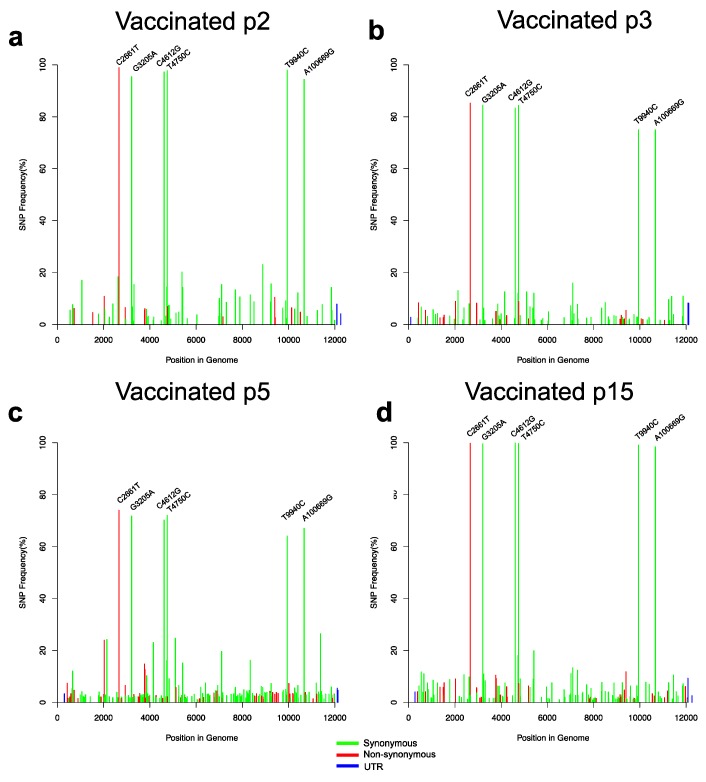
SNP frequency plots of the vaccinated pigs following the virus challenge determined following RT-PCR. SNPs with a frequency above 20% are indicated for each sample type as follows: (**a**) Serum (PID5) from pig p2 previously vaccinated with vR26E2gif. (**b**) Serum (PID5) from pig p3 previously vaccinated with vR26_E2gif. (**c**) Serum (PID5) from pig p5 vaccinated with vR26_E2gif and (**d**) Serum (PID5) from pig p15 vaccinated with vR26. The red, green, and blue colours of the bars represent non-synonymous, synonymous, and untranslated region (UTR) mutations, respectively.

**Figure 5:**
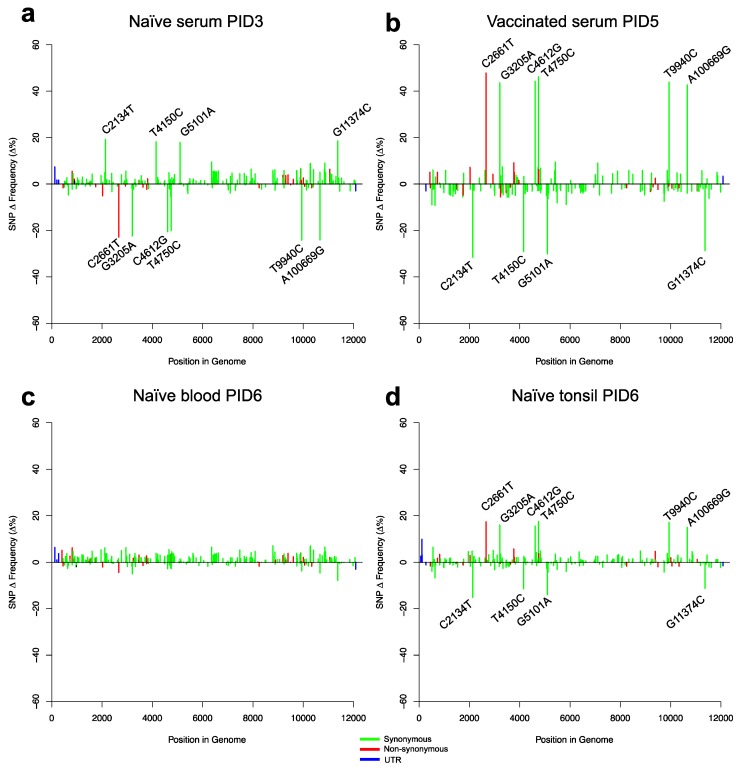
Relative SNP frequency distributions in the challenge virus depicted as the mean frequency change of SNPs for the vaccinated and naïve groups compared to the inoculum as Δ%. SNPs with large changes in frequency are labelled on the plots. Samples were: (**a**) Serum (PID3) from naïve pigs. (**b**) Serum (PID5) from vaccinated pigs. (**c**) Blood (PID6) from naïve pigs. (**d**) Tonsils (PID6) from naïve pigs. The red, green, and blue colours of the bars represent non-synonymous, synonymous, and untranslated region (UTR) mutations, respectively.

To investigate evolutionary changes in the virus population after challenge, we generated sequence data from both the naïve and vaccinated animals. From the naïve group, we sequenced virus extracted from serum samples at PID3 and PID6, as well as from blood and tonsil samples obtained at PID6 (Figure 3). In the vaccinated group, viral loads after challenge were low but we obtained NGS data determined following RT-PCR from serum samples obtained at PID5 from three vR26_E2gif vaccinated pigs and from one pig vaccinated with the vR26 (Figure 4a–d). This NGS data represented exclusively the challenge virus population since vaccine virus is not amplified by this RT-PCR. Figure 3 and Figure 4 show the frequencies of all SNPs found in the viral populations from naïve and vaccinated animals, while Figure 5 shows the change in SNP frequencies between the inoculum and samples from naïve or vaccinated animals. 

In general, virus populations in both naïve and vaccinated animals can be seen to be quite heterogeneous with numerous low-frequency (5–10%) SNPs being present. Viral populations in naïve animals had more of these low-frequency SNPs than populations in vaccinated animals (also see the investigation of nucleotide diversity below). Closer analysis showed that virus in vaccinated animals contained non-synonymous SNPs that were not found in the inoculum above the detection limit, suggesting a process of mutation followed by positive selection (Table 1). Thus, novel SNPs were seen in the Npro p7 and NS2 coding sequences (Table 1), but none of these changes were observed in the serum samples from naïve pigs. Several of the low-frequency SNPs found in the sera from vaccinated animals were also detected in the tonsils from the naïve pigs although at lower frequencies (Table 1).

Focusing on the more frequent SNPs, we found that the same 10 high-frequency SNPs that were present in the inoculum were also present at high frequency in serum and blood from naïve animals. In vaccinated animals, however, only 6 of these 10 SNPs, which included the non-synonymous mutation C2661T, were present at high frequency (Figure 5b; Table 1). Closer analysis of the changes in SNP frequencies between inoculum, and naïve or vaccinated animals, showed that, in naïve animals, 4 of the 10 high-frequency SNPs increased in frequency, while the remaining six had decreased. The pattern in vaccinated animals was exactly the opposite; in these animals the set of four SNPs had gone down in frequency, essentially disappearing from the population, while the frequency of the remaining six SNPs had gone up. Interestingly, tonsil samples from naïve animals showed the same pattern of SNPs as the vaccinated animals (i.e., the same six SNPs increased in frequency).

**Figure 6:**
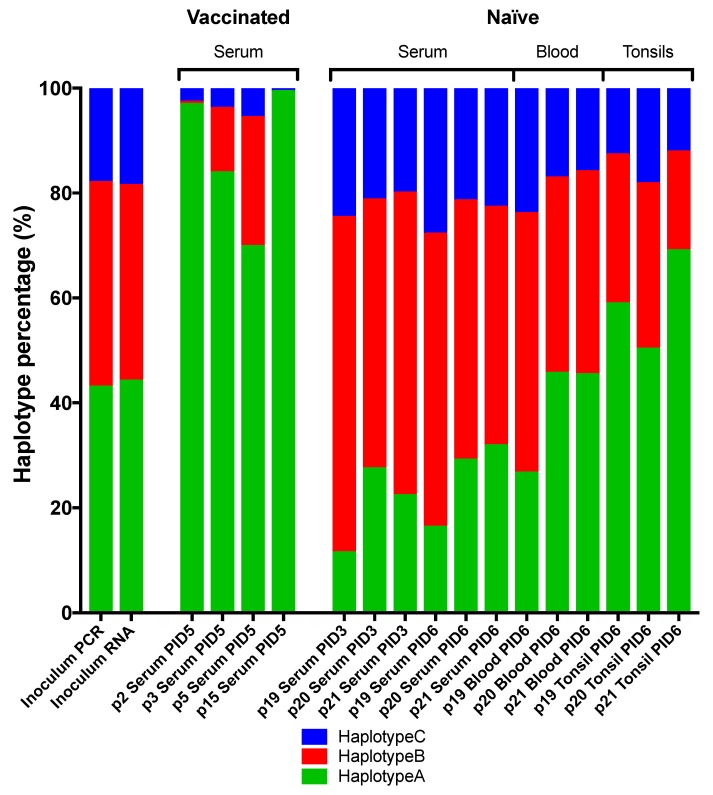
Haplotype distribution in virus populations of all samples across the complete ORF. Stacked bar diagram showing the percentage distribution of haplotype A (C2661T (aa S763L), G3205A, C4612G, T4750C, T9940C, and A10669G), B (C2134T, T4150C, G5101A, and G11374C), and C (Koslov consensus), respectively. Haplotype distribution was calculated for each sample as the average of all SNPs constituting the haplotype A or B and summed. The remaining percentage, subtracted from 100%, was inferred to constitute haplotype C.

These results strongly suggested that at least two major haplotypes were present in the original challenge inoculum. One of these (termed “haplotype A”) contains six SNPs compared to the Koslov consensus reference (C2661T, G3205A, C4612G, T4750C, T9940C, and A10669G) of which the C2661T change resulting in the S763L substitution is the only non-synonymous mutation. The second (“haplotype B”) contains four synonymous SNPs compared to the reference (C2134T, T4150C, G5101A, and G11374C). Since the SNP frequencies of haplotype A and B do not add up to 100%, and since we observed no other major SNPs compared to the Koslov reference, a third haplotype (“C”), identical to the reference consensus sequence, can be inferred to make up the remainder of the virus population. Figure 6 and Figure S2 summarise the distribution of these three haplotypes in the different groups of animals and sample types. It can be seen that the haplotype composition in the inoculum is about 40% A, 40% B, and 20% C. After challenge with this inoculum, in the vaccinated animals, haplotype C (the Koslov consensus) essentially disappeared, while haplotype A, which includes the non-synonymous SNP, became the dominant one. Indeed, in two of the four investigated animals, haplotype B disappeared almost completely, while in the other two it decreased to 10%–20% of the total. In the naïve animals, we found that the haplotype-distribution was different in different types of samples, suggesting that the haplotypes may be associated with cell- or tissue-tropism. Thus, tonsil samples from naïve animals were found to be similar to serum samples from vaccinated animals in that haplotype A dominated, whereas serum samples displayed the opposite pattern, with a decrease in the level of haplotype A and an increased proportion of B. Whole-blood was found to be similar to the original inoculum.

The haplotypes described above were inferred based on frequencies (and changes of frequencies) of individual SNPs obtained from short sequencing reads. This type of data contains limited information about the linkage of genetic variants. Thus, in order to more directly investigate which haplotypes were present, we cloned a large cDNA fragment covering the first half of the genome, derived from a tonsil sample from one of the naïve pigs (p19), that spanned 6 out of the 10 high-frequency SNPs (at nt 2661, 3205, 4150, 4612, 4750, and 5101). By sequencing 15 separate clones, it was possible to see directly which SNPs were linked to individual virus genomes (Table 2). The sequencing results confirmed the presence of the two major inferred haplotypes A (C2661T, G3205A, C4612G, and T4750C) and B (T4150C and G5101A) in 47% and 27% of the clones, respectively (Table 2). Furthermore, the inferred haplotype C, without any of the six SNPs, was indeed found to be present in the rest of the clones (Table 2).

### 3.4. Loss of CSFV Population Heterogeneity and Targeted Positive Selection was Observed in the Vaccinated Pigs

**Figure 7:**
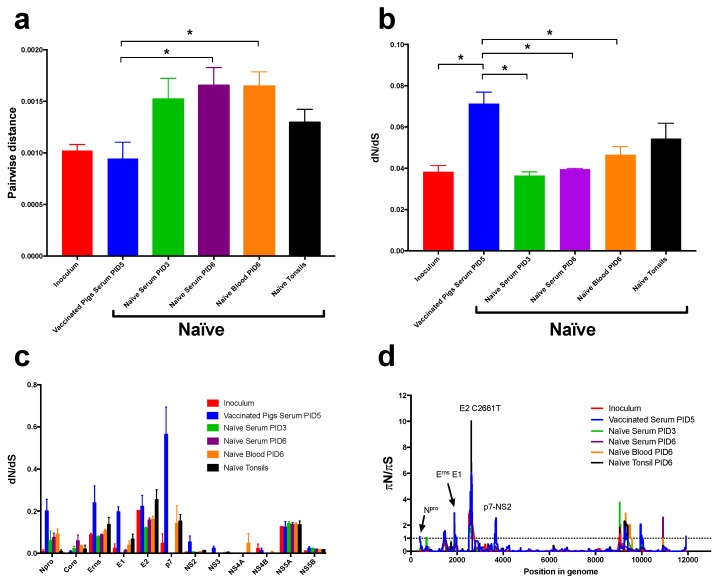
Population diversity derived by selection analysis of deep sequencing data. Panel (**a**) shows the pair-wise genetic distance plotted for each individual sample type across the complete ORF. Means ± SEM are shown for biological replicates (*n* = 3 and *n* = 4) for the naïve and vaccinated groups, respectively, and technical replicates for the inoculum (*n* = 2). The T-test was applied to determine significance differences between the vaccinated group compared to blood, tonsils, and serum (PID3 and PID6) of the samples from naïve pigs (*p* = 0.0242*, *p* = 0.1649, *p* = 0.070*, *p* = 0.0304*), respectively, and to the challenge inoculum (*p* = 0.7598) with * indicating significant values. Panel (**b**) depicts the dN/dS ratio plotted for each individual sample type for the total ORF. The T-test was applied, as above, to determine significant differences between the vaccinated group compared to blood, tonsils, and serum (PID3 and PID6) of the naïve samples (*p* = 0.0193*, *p* = 0.1165, *p* = 0.0033*, *p* = 0.0045*), respectively, and to the inoculum (*p* = 0.0172*). Panel (**c**) shows the dN/dS ratios plotted for each individual protein-coding sequence from the inoculum, vaccinated, and naïve groups. Panel (**d**) shows the πN/πS ratios plotted as lines representing a 150 nt sliding window with the x-axis representing the position in the genome.

Population heterogeneity was quantified by calculating “nucleotide diversity”, i.e., the average pairwise differences between sequences. The samples collected from the naïve pigs post-challenge all showed increased virus diversity compared to the inoculum (Figure 7a). Vaccinated pigs, however, were found to harbour viral populations that were much less heterogeneous than those in the naïve animals, indicating differences in selective pressure in the two groups of animals.

Further analysis of positive selection in the individual samples was performed by estimating the dN/dS and πN/πS ratios (where N and S indicate non-synonymous and synonymous changes respectively). In general, dN/dS ORF ratios were well below 1 for all groups indicating that averaged over all codons the viral proteins are under negative selection. In vaccinated animals, and in the tonsil samples from naïve pigs, dN/dS was increased (but still well below 1), compared to the inoculum (Figure 7b). This can be caused by relaxed negative selection, or the presence of some codons being under positive selection (e.g., due to selective pressure to escape from the immune response). We also observed differences in the viral sequences encoding the Npro, Erns, E1, and p7 proteins between the vaccinated pigs and the naïve pigs (Figure 7c). When comparing the different sample types within the naïve animals, we found an increase in the dN/dS ratio for the E2 and p7 protein-coding sequences in the tonsils.

To further investigate regions of the genome under positive selection, a sliding window analysis was performed (Figure 7d) with the non-synonymous change C2661T producing the highest πN/πS ratio in all groups.

This analysis revealed distinct sites in the virus samples from the vaccinated pigs in the Npro, E1, and p7 to NS2 junction coding sequences compared to the naïve pigs pointing to positive selection acting on these parts of the genome. One site displaying a particularly high πN/πS ratio was observed within the Npro coding region; this is due to the non-synonymous SNP A425G (resulting in the M18V substitution in the N-terminus of the protein). In the Erns coding region, there were non-synonymous changes including several low-frequency SNPs that, in combination, and together with the low number of synonymous SNPs, resulted in the observed peak in the πN/πS ratio. Among the coding changes, the SNP T2024C (F551L) gave rise to the elevated πN/πS ratio within the E1 coding region; this change was detected in the vaccinated pigs but was also found in the inoculum but at a lower frequency. The prominent peak in the πN/πS ratio located in the p7 protein-coding sequence is due to the non-synonymous SNP A3765G (K1131R); this was not found in the inoculum while the nearby G3782A (G1137S) change within the coding region for the NS2 protein could be detected in the inoculum.

## 4. Discussion

The chimeric pestivirus, vR26_E2gif, represents a C-strain based marker vaccine candidate against CSF [10]. A vaccination/challenge experiment was performed to assess the vaccine properties of this chimeric vaccine in comparison to the parental C-strain vaccine vR26. Our results demonstrated that vR26_E2gif could efficiently protect pigs against disease with only transient fever being observed in some vaccinated animals after challenge with the highly virulent CSFV Koslov. Notably, no transmission was observed between the challenged animals and the sentinels. In addition, vaccination using the vR26_E2gif could be efficiently discriminated from the use of the C-strain vaccine vR26 and wild-type CSFV using the CSFV E2-specific antibody ELISA. This is an important feature of this marker vaccine prototype. However, low levels of the challenge virus were observed in blood samples from the vR26_E2gif vaccinated pigs between PID3 and PID10. Furthermore, the challenge virus was also detected in pigs previously vaccinated with vR26, but to a lower extent. Therefore, we decided to investigate the nature of the challenge virus present in the animals under the selective pressure imposed by vaccination. Interference from the vaccine virus in the sequence data could be ruled out since no vaccine virus was detected at any time after vaccination and the RT-PCR approach specifically targeted the challenge virus population. Detailed SNP and dN/dS analysis allowed us to discover patterns of change within the Koslov virus in the vaccinated animals that were not seen in the naïve animals. In addition, we detected several amino acid residues under positive selection in the vaccinated animals and an apparent haplotype tropism selection in the naïve animals. 

Thus, the main focus of this study was the analysis of the molecular evolution of the virus populations within infected pigs based on deep sequencing data. Deep sequencing of the inoculum and samples from naïve and vaccinated animals showed that in all cases the viral populations were heterogeneous, consisting of a cloud of related viral sequences with many low-frequency SNPs as well as a few high-frequency variants. Closer analysis of changes in these SNP frequencies allowed us to infer that the viral populations consisted of three main haplotypes (A, B, and C) that were closely related. The first of these, haplotype A, had six SNPs compared to the Koslov reference sequence and included a non-synonymous change at nt position 2661 (C2661T, corresponding to the amino acid substitution S763L). The second one, haplotype B, contained four SNPs—all synonymous—compared to the reference. The last one, haplotype C, was identical to the reference. The nature of these haplotypes was confirmed by sequencing cloned fragments from a tonsil sample that covered 6 of the 10 SNPs in single molecules.

Analysis of the composition of haplotypes in different samples from naïve and vaccinated animals after viral challenge showed that haplotype A, which contains the non-synonymous change C2661T (S763L) in the coding region for the E2 protein was selected for in the vaccinated pigs and also in the tonsils of the naïve pigs. This suggests that haplotype A replicates more efficiently in the tonsils and that this haplotype may be partially resistant to the immune response induced by vaccination, resulting in its near fixation as observed in serum from vaccinated pigs. The virus in the blood from infected naïve animals had a haplotype composition that was very similar to that found in the original challenge inoculum, while virus in the serum from these same animals showed an increase in haplotype B relative to A (i.e., the opposite pattern from that seen in vaccinated animals and in tonsils). The observation that the haplotype composition of the virus in whole blood of naïve animals is the same as that in the virus inoculum suggests that diversity is being actively maintained as has previously been observed for other highly virulent CSFV populations [5]. Heterogeneity, in itself, is not necessary for high virulence as can be seen from the fact that virus rescued from a cDNA clone had similar virulence as the wild-type inoculum [7]. It does, however, seem that tissue tropism and immune escape play a role in determining haplotype composition and population heterogeneity as seen for other viruses [8,36]. Indeed, our data suggest that haplotypes A and B, respectively, replicate most efficiently in two different tissues and this could be why both are maintained in the naïve pigs.

The maintenance of the three haplotypes in the naïve pigs can be described as three peaks within the fitness landscape around which there is a cloud of related suboptimal viruses. Most of the low-frequency SNPs are removed by selection constantly, while the three main haplotypes may be maintained by each having its own tissue in which it replicates well. However, vaccination shifts the fitness landscape towards a higher fitness for haplotype A [37,38].

It seems relevant that amino acid residue 763, which is altered by one of the SNPs in haplotype A, is situated within a putative epitope on the E2 surface protein of the virus [39]. Using the known 3D structure of the E2 protein from bovine viral diarrhoea virus [40] as a model for the Koslov E2 protein, we found that residue S763 (amino acid 74 in the E2 protein) is situated on the surface of domain 1 in the antigenic region B/C, suggesting a role in cell entry and immunogenicity. This region has also been proposed to be involved in membrane fusion inside the endosomes that is facilitated by E1 at low pH [40,41]. Both the CSFV C-strain and BDV Gifhorn have E2 proteins that contain leucine (L) at position 763, while Koslov contains serine (S) at this site. It has previously been observed that the adjacent amino acid, at position 764, could change from the L present in Koslov to the P [7] also found in both the C-strain and Gifhorn. This indicates that the combination of these two positions is important and this could explain why the vaccine constructs containing E2 Gifhorn as well as the E2 C-strain each created an immune pressure to select for haplotype A of the Koslov challenge virus. However, further studies using reverse genetics and in vitro and in vivo characterization of this epitope are needed to completely understand these phenomena. 

Virus from the serum of vaccinated pigs was found to have a higher dN/dS ratio than virus in naïve pigs. This is consistent with an increased positive selective pressure being exerted by the immune system in the vaccinated pigs driving adaptation, which leads to a slightly higher average ratio although most codons are still under purifying selection (dN/dS < 1). We also found that viral populations in vaccinated pigs were less diverse than those in naïve pigs; this again is probably an effect of the pressure from the immune system. Sliding window analysis revealed that certain residues within the Core, E^rns^, and p7 proteins were under positive selection especially within the p7 region. In addition, this analysis pointed towards the increase in dN/dS being the result of individual, positively selected codons that emerged in response to the pressure from the prior vaccination. The role of these residues could be interesting for future investigations of immune escape mechanisms. In addition, within the naïve animals, virus present within tonsils displayed significantly higher levels of positive selection for amino acid residues within the E2 and p7 proteins compared to the virus present in blood and serum from the same animals. 

We suggest that these analyses can benefit future virus vaccine studies by allowing identification of adaptive mutations and sites important for potential immune escape of the challenge virus. The knowledge gathered here may thus be useful for the production of improved live attenuated vaccines and lead to an enhanced understanding of the adaptive potential of a virus.

## Figures and Tables

**Table 1:** SNPs in vaccinated animals compared to naïve animals.

Protein	Nucleotide Position	Inoculum (nt)	Variant (nt)	Vaccinated (serum) SNP Frequency %	Vaccinated (serum) SNP Frequency Δ %	Naïve (serum) SNP Frequency %	Naïve (tonsils) SNP Frequency %	SNP Effect	Comments
**N^pro^**	425	A	G	5.1	5.1	-	-	M18V	*
	547	A	G	6.1	6.1	-	6.4	-	*
	658	C	T	7.7	3.0	-	3.6	-	
	725	A	G	5.1	5.1	-	1.7	I118V	*
	814	C	T	3.1	0.6	1.3	1.5	-	
**C**	1057	T	C	8.9	5.9	2.5	3.8	-	
**E^rns^**	1774	A	G	4.6	4.6	-	1.3	-	*
**E1**	2024	T	C	13.8	7.2	1.52	9.4	F551L	
	2395	C	T	6.6	2.6	0.7	5.8	-	
**E2**	2617	T	C	9.5	5.8	2.8	5.5	-	
	2650	T	C	3.9	3.9	-	1.5	-	*
	2661	C	T	89.3	47.8	18.6	58.9	S763L	
	2935	G	A	6.7	4.2	1.65	3.1	M854I	
	3205	G	A	87.7	43.6	21.7	60.1	-	
	3244	A	G	6.5	3.5	0.6	6.4	-	
	3310	G	A	7.4	7.4	-	-	-	*
**p7**	3765	A	G	9.1	9.1	-	5.6	K1131R	*
**NS2-3**	3782	G	A	8.1	5.1	0.7	5.1	G1137S	
	3853	G	A	6.8	3.8	1.1	3.2	-	
	3907	G	A	2.9	2.9	-	-	-	*
	3931	T	C	1.8	1.9	-	2.0	-	*
	3965	G	A	1.5	1.5	-	-	V1198M	*
	4168	A	G	3.3	3.3	-	2.4	-	*
	4612	C	G	87.7	44.2	23.0	59.0	-	
	4729	A	G	15.2	7.2	2.7	9.2	-	
	4750	T	C	88.3	46.2	22.1	59.6	-	
	4760	G	A	6.2	6.2	-	3.6	V1463I	*
	4828	T	C	6.8	6.8	1.45	4.7	-	
	5248	A	G	6.0	2.2	3.0	3.4	-	
	5389	C	T	9.2	5.7	0.7	3.7	-	
	5416	T	C	15.4	9.5	1.9	9.3	-	
	6028	G	A	1.6	1.6	-	-	-	*
	7006	G	A	5.7	4.0	0.6	2.9	-	
	7021	T	C	5.1	1.9	0.6	5.7	-	
	7099	A	G	16.2	9.0	3.3	9.7	-	
**NS4A**	7304	T	C	6.9	3.4	0.7	1.4	-	
	7318	A	T	1.8	0.1	0.6	2.2	-	
**NS4B**	7696	T	C	7.2	4.5	2.3	4.0	-	
	7888	T	C	5.6	5.6	1	2.9	-	
	8341	C	T	9.5	5.9	1.4	5.0	-	
**NS5A**	8500	C	T	6.0	4.2	0.6	3.4	-	
	8878	G	A	9.7	5.8	1.2	3.7	-	
	9241	A	T	8.3	4.7	1.9	3.4	-	
	9396	A	G	7.8	2.5	9.2	9.9	K3008R	
	9871	G	A	5.8	5.9	1.4	3.6	-	
	9901	T	C	1.6	1.6	-	-	-	*
**NS5B**	9940	T	C	85.7	43.9	17.6	59.2	-	
	10259	T	C	4.8	4.8	-	-	-	*
	10402	C	T	6.9	4.3	1.3	3.1	-	
	10669	A	G	85.3	42.6	18.6	57.7	-	
	11242	T	C	5.7	5.7	-	-	-	*
	11452	C	T	5.4	2.3	-	6.1	-	
	11849	C	T	7.4	5.0	2.3	3.5	-	
	11872	A	G	6.5	3.5	-	4.0	-	
**3′ UTR**	12083	C	T	6.3	3.3	-	1.4	-	

SNP frequencies are depicted as the average within each group. Δ% is the average SNP frequency compared to the inoculum frequency. * SNPs in vaccinated animals only and not in naïve animals nor in virus inoculum. Grey shading represents SNPs present in haplotype A.

**Table 2:** Identification of virus haplotypes.

cDNA *	C2661T	G3205A	T4150C	C4612G	T4750C	G5101A	Haplotype
	(S763L)						
P19_1	+	+	-	+	+	-	A
P19_2	+	+	-	+	+	-	A
P19_3	+	+	-	+	+	-	A
P19_4	-	-	+	-	-	+	B
P19_5	+	+	-	+	+	-	A
P19_6	+	+	-	+	+	-	A
P19_7	-	-	+	-	-	+	B
P19_8	-	-	+	-	-	+	B
P19_9	-	-	-	-	-	-	C
P19_10	-	-	+	-	-	+	B
P19_11	-	-	-	-	-	-	C
P19_12	-	-	-	-	-	-	C
P19_13	-	-	-	T **	-	-	C
P19_14	+	+	-	+	+	-	A
P19_15	+	+	-	+	+	-	A

* Individual cDNA clone derived from p19 tonsil. ** C4612T. Each (-) indicates that the variant was not detected in the cDNA and (+) indicates that the variant was detected.

## Supplementary Materials

The following are available online at https://www.mdpi.com/1999-4915/11/10/932/s1, Figure S1: CSFV E2 specific antibodies detected by ELISA in naïve pigs or vaccinated pigs, Figure S2: Haplotype distribution in virus populations of pooled samples analysed across the complete ORF, Table S1: RNA extraction and RT-PCR amplification from tonsils, Table S2: Samples sequenced, NGS platform, and data analysis.
