# Supplementary material for: Virus Adaptation and Selection Following Challenge of Animals Vaccinated against Classical Swine Fever Virus
Source: Viruses. 2019 Oct 10;11(10):932. doi: 10.3390/v11100932 (PMC6833067; doi:10.3390/v11100932)
Supplement: Supplementary file 1 [file viruses-11-00932-s001.pdf]

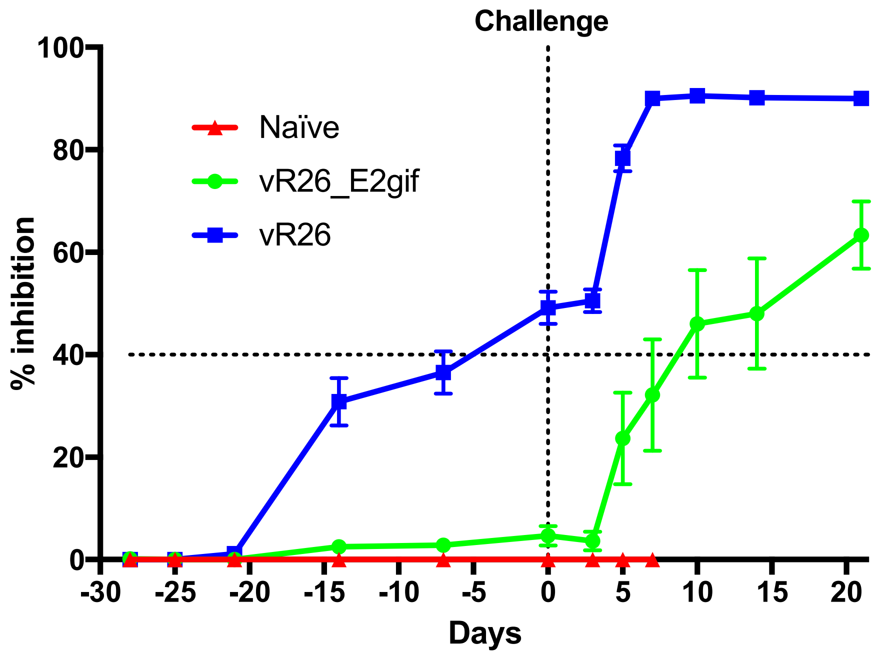

Figure S1. CSFV E2 specific antibodies detected by ELISA in naïve pigs or vaccinated pigs. Naïve pigs or pigs vaccinated with vR26, or vR26_E2gif (as indicated) were challenged with the highly virulent CSFV strain "Koslov". The graph shows the (mean ± SEM values) of CSFV E2 specific antibodies detected by ELISA (PrioCHECK CSFV Ab 2.0, Prionics) with a positive cut-off at 40% inhibition shown as a horizontal dashed line.

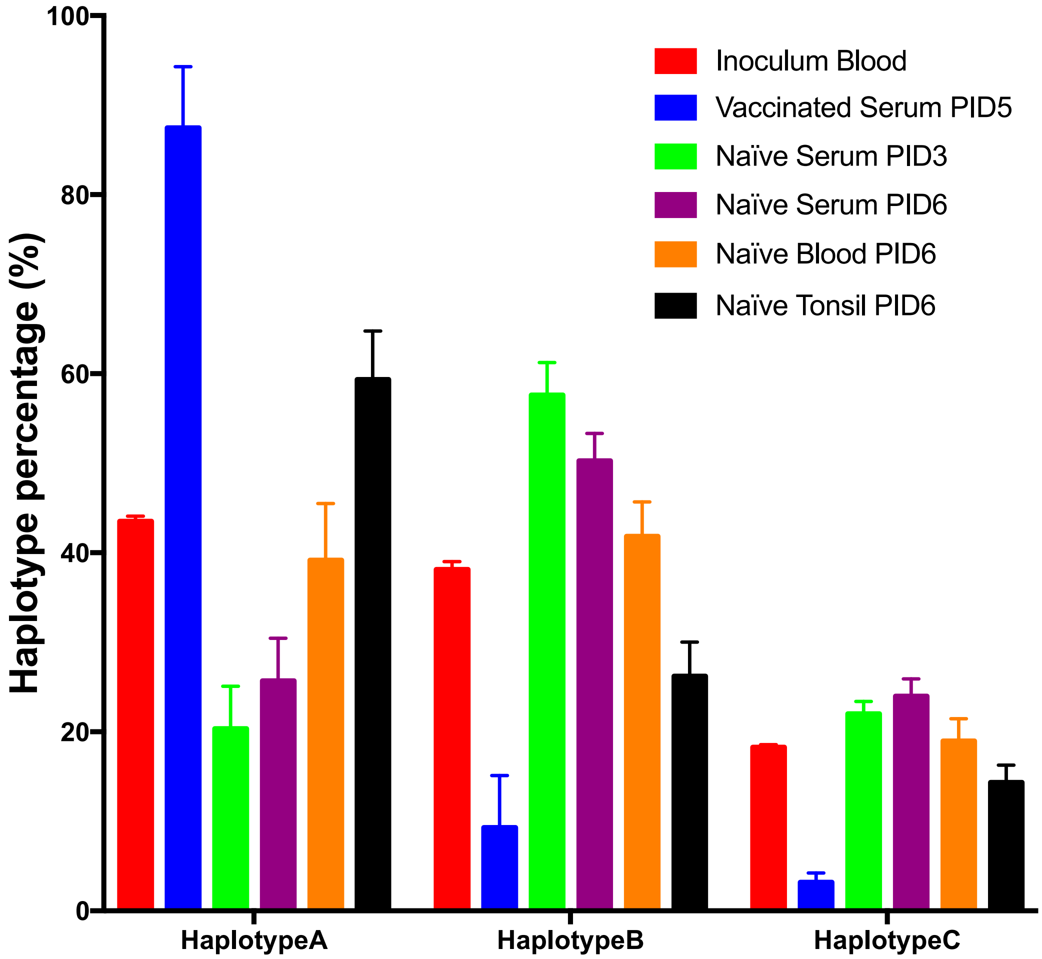

Figure S2. Haplotype distribution in virus populations of pooled samples analysed across the complete ORF. The graphs show the mean ± SEM values. Bar diagram showing the percentage distribution of haplotype A (C2661T (aa S763L), G3205A, C4612G, T4750C, T9940C and A10669G), B (C2134T, T4150C, G5101A and G11374C) and C (Koslov consensus), respectively. Haplotype distribution was calculated for each sample, as the average of all SNPs constituting the haplotype A or B, and summed. The remaining percentage, subtracted from 100%, was inferred to constitute haplotype C.

Table S1. RNA extraction and RT-PCR amplification from tonsils

RNA (Eluate)	1	2	3	4	5
RNA (ng/µl)	2719	1700	208	19	2
ct	19.9	20.8	19.7	21.2	22.5
RT-PCR Band intensity*	(+)	+	+++	+++	++

*Band intensity was visualized on a 1% agarose gel and the number of + reflects the intensity.

Table S2. Samples sequenced, NGS platform and data analysis.

Group	Material sequenced	Pig no.[1]	Vaccine	PID	RT-PCR[2]	NGS input	NGS platform
Inoculum	Blood	NA	NA	0	-	RNA	FLX
	Blood	NA	NA	0	F	cDNA	FLX
Vaccinated	Serum	p2	vR26E2gif	5	H	cDNA	FLX
	Serum	p3	vR26E2gif	5	H	cDNA	PGM
	Serum	p5	vR26E2gif	5	H	cDNA	PGM
	Serum	p15	vR26	5	H	cDNA	PGM
Naïve	Serum	p19	NA	3	H	cDNA	PGM
	Serum	p19	NA	6	-	RNA	FLX
	Blood	p19	NA	6	H	cDNA	PGM
	Tonsil	p19	NA	6	F	cDNA	PGM
	Serum	p20	NA	3	H	cDNA	PGM
	Serum	p20	NA	6	-	RNA	FLX
	Blood	p20	NA	6	H	cDNA	PGM
	Tonsil	p20	NA	6	F	cDNA	PGM
	Serum	p21	NA	3	H	cDNA	FLX
	Serum	p21	NA	6	-	RNA	FLX
	Blood	p21	NA	6	F	cDNA	FLX
	Tonsil	p21	NA	6	F	cDNA	PGM

[1]NA: Not applicable. [2]F: Full-length RT-PCR amplicon; H: 2 x half-length RT-PCR amplicons
